# Association between renal function impairment and multivessel involvement in patients with acute ST-elevation myocardial infarction

**DOI:** 10.18632/aging.103299

**Published:** 2020-05-20

**Authors:** Rei-Yeuh Chang, Han-Lin Tsai, Malcolm Koo, How-Ran Guo

**Affiliations:** 1 Division of Cardiology, Department of Internal Medicine, Ditmanson Medical Foundation Chia-Yi Christian Hospital, Chiayi City, Taiwan; 2 Chung Jen Junior College of Nursing, Health Sciences and Management, Chiayi County, Taiwan; 3 Min-Hwei Junior College of Health Care Management, Tainan City, Taiwan; 4 Graduate Institute of Long-term Care, Tzu Chi University of Science and Technology, Hualien City, Taiwan; 5 Dalla Lana School of Public Health, University of Toronto, Toronto, Canada; 6 Department of Environmental and Occupational Health, National Cheng Kung University, Tainan City, Taiwan; 7 Department of Occupational and Environmental Medicine, National Cheng Kung University Hospital, Tainan City, Taiwan

**Keywords:** acute myocardial infarction, chronic kidney disease, multivessel disease

## Abstract

The aim of this study was to evaluate the association between acute ST-elevation myocardial infarction (STEMI) involving multivessel and the severity of renal function impairment. We reviewed medical records of patients with acute STEMI admitted to a regional teaching hospital in southern Taiwan between March 1999 and October 2013. A total of 1215 patients who underwent coronary angiography were included. Multiple logistic regression analysis showed that multivessel involvement (at least two) with significant stenosis was significantly associated with stage 4 chronic kidney disease (adjusted odds ratio [aOR]=2.14, 95% confidence interval [CI]=1.09–4.20) and stage 5 chronic kidney disease (aOR=2.35, 95% CI=1.13–4.89), adjusting for age, sex, type 2 diabetes mellitus, hyperlipidemia, and systolic blood pressure at admission in patients with acute STEMI. In addition, multivessel total occlusion was significantly associated with stage 4 chronic kidney disease (aOR=3.68, 95% CI=1.27–10.70) and stage 5 chronic kidney disease (aOR=3.43, 95% CI=1.08–10.82), adjusting for heart rate at admission and systolic blood pressure at admission in patients with acute STEMI. In conclusion, severe renal function impairment was significantly associated with multivessel significant stenosis and multivessel total occlusion in patients with acute STEMI.

## INTRODUCTION

Chronic kidney disease (CKD) is a serious health problem worldwide, and cardiovascular disease is a common cause of death in the patients. The burden of CKD increases with the aging of population and the growing number of patients with diabetes mellitus and hypertension. The prevalence of moderate to severe CKD (stages 3 to 5) has increased from 6% to 10% in recent years [1]. Some observational studies have shown that a reduced glomerular filtration rate (GFR) and an increased albuminuria level were both independently associated with an increased risk of all-cause and cardiovascular mortality in the general population [2]. Many studies that enrolled patients with known risk factors for cardiovascular disease, such as hypertension and diabetes, or preexisting cardiovascular disease have shown that the presence or development of various degrees of renal dysfunction was independently associated with cardiovascular events [3, 4]. A nationwide study of 130,099 patients with myocardial infarction in the United States revealed that an elevated creatinine level was a significant predictor of mortality risk and underutilization of cardiovascular prevention therapies, including aspirin, beta-blockers, thrombolytic therapy, angiography, and angioplasty during the period of hospitalization [5]. Another study showed a strong dose-response relationship between renal function and in-hospital mortality as well as post-discharge survival at 1, 3, and 5 years in patients with acute myocardial infarction (AMI) [6]. In addition, patients with CKD who received less aggressive treatment during hospitalization for AMI showed substantially higher mortality rates [6, 7].

Previous studies indicated that the severity of atherosclerosis of the coronary artery was correlated with the declining of GFR in patients with acute coronary syndrome [8]. In AMI, both the number of coronary arteries with significant stenosis and that of those with total occlusion can affect the cardiologist’s decision on the modality of revascularization, i.e., medical therapy, primary percutaneous coronary intervention (PCI), or coronary artery bypass grafting (CABG). CKD not only is an independent risk factor for cardiovascular disease, it is also frequently associated with other risk factors, such as old age, hypertension, and diabetes. The morbidities and complications of CKD patients during admission of AMI are generally higher than those of patients with normal renal function.

The risk of post procedural acute kidney injury is a concern in patients with CKD. Therefore, the choice of treatment modality for acute ST-elevation myocardial infarction (STEMI) in CKD patients is a challenge to cardiologists. Therefore, the aim of this study was to evaluate the associations between the severity of renal impairment and the risk of having multiple (at least 2) coronary arteries with significant stenosis or total occlusion in patients with acute STEMI.

## RESULTS

Of the 1,815 acute STEMI patients identified during the study period, 1,365 received coronary angiography, and 1,215 with no missing data, except hemoglobin (100 patients with missing data) and proteinuria (250 patients with missing data), were included in the analyses. The mean age of the patients was 62.0 years, and 78.2% of them were male. The demographic and clinical characteristics of the study patients are shown in Table 1. The distribution of the number of vessels with significant stenosis was significantly different among patients at different CKD stages (*P* = 0.001). However, the distribution of the number of vessels with total occlusion was not significantly different among patients at different CKD stages (*P* = 0.243). In addition, the remaining variables were significantly different among the five CKD stages except for peak creatine kinase, peak creatine kinase-MB, peak activated partial thromboplastin time, infarct location, infarct-related artery, and four types of complications (death, ventricular septal rupture, free wall rupture, and ventricular tachycardia).

**Table 1:** Demographic and clinical characteristics of study patients (N = 1215).

**Variable**	**Stage of chronic kidney disease Number (%) or mean (SD)**					***P***
	**Stage 1 153 (12.6)**	**Stage 2 542 (44.6)**	**Stage 3 398 (32.8)**	**Stage 4 69 (5.7)**	**Stage 5 53 (4.4)**	
Age (year)	51.5 (2.4)	60.1 (11.2)	67.0 (10.6)	71.0 (11.7)	65.8 (13.1)	<0.001
Sex						<0.001
Male	128 (83.7)	443 (81.7)	303 (76.1)	41 (59.4)	35 (66.0)	
Female	25 (16.3)	99 (18.3)	95 (23.8)	28 (40.6)	18 (34.0)	
Smoking	84 (54.9)	262 (48.3)	148 (37.2)	23 (33.3)	13 (24.5)	<0.001
Hypertension	64 (41.8)	274 (50.6)	224 (56.3)	49 (71.0)	39 (73.6)	<0.001
Diabetes mellitus	62 (40.5)	196 (36.2)	162 (40.7)	34 (49.3)	30 (56.6)	0.017
Hyperlipidemia	84 (54.9)	275 (50.7)	169 (42.5)	20 (29.0)	10 (18.9)	<0.001
Hemoglobin (g/dL)	14.8 (1.7)	14.6 (1.8)	14.0 (2.1)	13.4 (2.2)	12.3 (2.5)	<0.001
Proteinuria						<0.001
Nil	86 (72.3)	302 (67.6)	177 (56.9)	16 (30.8)	12 (33.3)	
Trace	18 (15.1)	107 (23.9)	85 (27.3)	21 (40.4)	6 (16.7)	
Positive	15 (12.6)	38 (8.5)	49 (15.8)	15 (28.8)	18 (50.0)	
Length of hospital stay (day)	5.9 (3.4)	6.1 (4.5)	7.7 (9.2)	8.5 (7.4)	10.6 (10.8)	<0.001
Body weight (kg)	69.0 (13.3)	67.8 (11.6)	64.4 (11.9)	64.4 (15.5)	63.1 (15.3)	<0.001
Systolic blood pressure at admission (mmHg)	138.0 (25.5)	133.4 (27.8)	131.0 (32.4)	122.8 (33.1)	128.1 (32.6)	0.004
Diastolic blood pressure at admission (mmHg)	96.3 (94.6)	79.8 (17.7)	77.0 (19.0)	73.3 (20.2)	71.8 (18.5)	<0.001
Heart rate at admission (beat/minute)	85.0 (18.2)	79.6 (19.7)	80.3 (22.3)	84.6 (20.8)	83.7 (27.5)	0.023
Left ventricular ejection fraction (%)	56.2 (43.0)	52.9 (12.8)	51.9 (13.1)	47.7 (14.0)	48.0 (12.9)	0.012
Peak creatine kinase (U/L)	2227 (2311)	2255 (2532)	2265 (2272)	2148 (2203)	3070 (5531)	0.273
Peak creatine kinase-MB (ng/mL)	268.2 (622.2)	214.8 (232.3)	221.9 (325.9)	197.2 (228.9)	223.4 (376.1)	0.497
Peak troponin I (ng/mL)	65.8 (94.2)	84.3 (141.1)	103.8 (188.6)	121.6 (258.3)	134.5 (307.7)	0.035
Peak activated partial thromboplastin time (second)	47.7 (42.7)	45.6 (29.7)	50.4 (37.1)	52.6 (41.2)	54.5 (43.7)	0.134
Creatinine (mg/dL)	0.94 (0.59)	1.17 (0.68)	1.46 (0.47)	2.37 (0.94)	6.38 (3.02)	<0.001
eGFR (mL/min/1.73 m^2^)	99.3 (66.1)	70.5 (13.1)	51.0 (11.6)	32.3 (19.1)	14.9 (18.8)	<0.001
Killip class						<0.001
I–II	140 (91.5)	468 (86.3)	299 (75.1)	34 (49.3)	27 (50.9)	
III–IV	13 (8.5)	74 (13.7)	99 (24.9)	35 (50.7)	26 (49.1)	
Infarct location						0.618
Anterior	88 (57.5)	277 (51.1)	202 (50.8)	36 (52.2)	23 (43.4)	
Inferior	62 (40.5)	246 (45.4)	182 (45.7)	31 (44.9)	27 (50.9)	
Lateral	3 (2.0)	12 (2.2)	7 (1.8)	1 (1.4)	3 (5.7)	
Posterior	0 (0)	7 (1.3)	7 (1.8)	1 (1.4)	0 (0)	
Infarct-related artery						0.623
Left anterior descending artery	85 (56.3)	269 (50.1)	195 (49.9)	37 (54.4)	21 (41.2)	
Right coronary artery	56 (37.1)	219 (40.8)	161 (41.2)	29 (42.6)	26 (51.0)	
Left circumflex artery	10 (6.6)	43 (8.0)	30 (7.7)	2 (2.9)	3 (5.9)	
Left main coronary artery	0 (0)	6 (1.1)	3 (0.8)	0 (0)	1 (2.0)	
Saphenous vein graft	0 (0)	0 (0)	2 (0.5)	0 (0)	0 (0)	
Number of vessels with significant stenosis						0.001
None	0 (0)	0 (0)	1 (0.3)	0 (0)	0 (0)	
One	74 (48.4)	225 (41.5)	129 (32.4)	17 (24.6)	13 (24.5)	
Two	39 (25.5)	153 (28.2)	118 (29.6)	17 (24.6)	16 (30.2)	
Three	40 (26.1)	164 (30.3)	150 (37.7)	35 (50.7)	24 (45.3)	
Number of vessels with total occlusion						0.243
None	61 (39.9)	217 (40.0)	160 (40.2)	22 (31.9)	16 (30.2)	
One	86 (56.2)	291 (53.7)	214 (53.8)	37 (53.6)	30 (56.6)	
Two	6 (3.9)	30 (5.5)	21 (5.3)	9 (13.0)	6 (11.3)	
Three	0 (0)	4 (0.7)	3 (0.8)	1 (1.4)	1 (1.9)	
Intervention						<0.001
TPA+PCI or CABG	24 (15.9)	87 (16.2)	61 (15.7)	3 (4.3)	4 (7.5)	
TPA only	4 (2.6)	16 (3.0)	9 (2.3)	1 (1.4)	2 (3.8)	
Primary PCI	73 (48.3)	286 (53.4)	192 (49.4)	37 (53.6)	22 (41.5)	
Scheduled PCI	32 (21.2)	95 (17.7)	87 (22.4)	14 (20.3)	9 (17.0)	
Medicine	9 (6.0)	22 (4.1)	11 (2.8)	5 (7.2)	1 (1.9)	
CABG	9 (6.0)	30 (5.6)	29 (7.5)	9 (13.0)	15 (28.3)	
Intra-aortic balloon pumping	4 (2.6)	14 (2.6)	29 (7.3)	9 (13.0)	17 (32.1)	<0.001
Complications						
Death	16 (10.7)	45 (8.7)	30 (7.9)	4 (6.0)	2 (4.3)	0.593
Ventricular septal rupture	0 (0)	0 (0)	2 (0.5)	1 (1.4)	1 (1.9)	0.056
Free wall rupture	0 (0)	2 (0.4)	6 (1.5)	0 (0)	1 (1.9)	0.147
Ventricular tachycardia	5 (3.3)	28 (5.2)	27 (6.8)	8 (11.6)	2 (3.8)	0.108
Complete atrioventricular block	2 (1.3)	24 (4.4)	24 (6.0)	8 (11.6)	7 (13.2)	0.001
Paroxysmal atrial fibrillation	5 (3.3)	25 (4.6)	27 (6.8)	6 (8.7)	8 (15.1)	0.010
Upper gastrointestinal bleeding	3 (2.0)	26 (4.8)	30 (7.5)	9 (13.0)	3 (5.7)	0.008
Stroke	1 (0.7)	0 (0)	1 (0.3)	2 (2.9)	3 (5.7)	<0.001
Acute kidney injury	1 (0.7)	10 (1.8)	20 (5.0)	10 (14.5)	11 (20.8)	<0.001

CABG: coronary artery bypass grafting; eGFR: estimated glomerular filtration rate; PCI: percutaneous coronary intervention; SD: standard deviation; TPA: tissue plasminogen activator.

Continuous variables are reported as mean (standard deviation) and categorical variables are reported as frequency (%).

An estimated glomerular filtration rate [eGFR] ≥ 90 mL/min/1.73 m^2^ is defined as stage 1, eGFR 60–89 mL/min/1.73 m^2^ is defined as stage 2, eGFR 30–59 mL/min/1.73 m^2^ is defined as stage 3, eGFR 15–29 mL/min/1.73 m^2^ is defined as stage 4, and eGFR < 15 mL/min/1.73 m^2^ is defined as stage 5.

A total of 100 patients (8.2%) and 250 patients (20.6%) had missing values in hemoglobin and proteinuria, respectively.

Furthermore, the number of vessels with total occlusion was significantly associated with the number of vessels with significant stenosis (Spearman's correlation coefficient = 0.23, *P* < 0.001). Among the 458 patients with significant stenosis in one coronary artery, 221 (48.2%) had no total occlusion, and the remaining 237 (51.8%) had one total occlusion. Among the 343 patients with significant stenosis of two coronary arteries, 134 (39.1%) had no total occlusion, 191 (55.7%) had one total occlusion, and the remaining 18 (5.2%) had two total occlusion. Finally, among the 416 patients with significant stenosis of three coronary arteries, 120 (29.1%) had no total occlusion, 230 (55.7%) had one total occlusion, 54 (13.8%) had two total occlusion, and the remaining 9 (2.2%) had three total occlusion.

Univariate logistic regression analyses showed that multivessel significant stenosis was significantly associated with older age, hypertension, type 2 diabetes mellitus, hemoglobin, systolic blood pressure at admission, and CKD status (stages 3, 4, and 5) in patients with acute STEMI (Table 2). Multiple logistic regression analysis further showed that multivessel significant stenosis was independently and significantly associated with age (adjusted odds ratio [aOR] = 1.02, 95% confidence interval [CI] = 1.01–1.03), male sex (aOR = 1.62, 95% CI = 1.20–2.19), type 2 diabetes mellitus (aOR = 1.70, 95% CI = 1.32–2.19), hyperlipidemia (aOR = 1.39, 95% CI = 1.08–1.78), systolic blood pressure at admission (aOR = 0.95 per 10 mmHg, 95% CI = 0.91–0.99) and CKD stages (Table 2). Specifically, using normal renal function or stage 1 CKD as the reference, the aOR was 2.14 (95% CI = 1.09–4.20) for stage 4 and 2.35 (95% CI = 1.13–4.89) for stage 5 (Table 2).

**Table 2:** Univariate and multiple logistic regression analyses of multivessel significant stenosis in patients with acute ST-elevation myocardial infarction.

**Variable**	**Univariate logistic regression**		**Multiple logistic regression**	
	**Odds ratio (95% confidence interval)**	***P***	**Odds ratio (95% confidence interval)**	***P***
Age (per year)	1.02 (1.01–1.03)	<0.001	1.02 (1.01–1.03)	<0.001
Sex (male versus female)	1.20 (0.91–1.58)	0.203	1.62 (1.20–2.19)	0.002
Smoking	1.03 (0.82–1.30)	0.791	–	–
Hypertension	1.30 (1.03–1.64)	0.028	–	–
Type 2 diabetes	1.66 (1.30–2.11)	<0.001	1.70 (1.32–2.19)	<0.001
Hyperlipidemia	1.15 (0.91–1.45)	0.245	1.39 (1.08–1.78)	0.010
Hemoglobin (per g/dL)	0.93 (0.88–0.99)	0.026	–	–
Proteinuria				
Nil	1.00			
Trace	1.06 (0.78–1.45)	0.691	–	–
Positive	1.18 (0.80–1.74)	0.411		
Body weight (per kg)	1.00 (0.99–1.01)	0.695	–	–
Systolic blood pressure at admission (per 10 mmHg)	0.95 (0.91–0.98)	0.007	0.95 (0.91–0.99)	0.011
Diastolic blood pressure at admission (per 10 mmHg)	0.98 (0.95–1.01)	0.271	–	–
Heart rate at admission (per 10 beat/minute)	0.97 (0.92–1.02)	0.261	–	–
Chronic kidney disease status				
Stage 1	1.00		1.00	
Stage 2	1.32 (0.92–1.89)	0.131	1.13 (0.78–1.66)	0.514
Stage 3	1.93 (1.32–2.82)	0.001	1.47 (0.97–2.24)	0.071
Stage 4	2.86 (1.52–5.40)	0.001	2.14 (1.09–4.20)	0.028
Stage 5	2.88 (1.43–5.81)	0.003	2.35 (1.13–4.89)	0.023

Furthermore, univariate logistic regression analyses showed that multivessel total occlusion was significantly associated with hemoglobin, systolic blood pressure at admission, heart rate at admission and CKD stages 4 and 5 (Table 3). Multiple logistic regression analysis further showed that multivessel total occlusion was independently and significantly associated with systolic blood pressure at admission (aOR = 0.92 per 10 mmHg, 95% CI = 0.85–1.00), heart rate at admission (aOR = 1.13 per 10 beats/min, 95% CI = 1.02–1.25) and CKD stages. Specifically, using normal renal function or stage 1 CKD as the reference, the aOR was 3.68 (95% CI = 1.27–10.70) for stage 4 and 3.43 (95% CI = 1.08–10.82) for stage 5 (Table 3).

**Table 3:** Univariate and multiple logistic regression analyses of multivessel total occlusion in patients with acute ST-elevation myocardial infarction

**Variable**	**Univariate logistic regression**		**Multiple logistic regression**	
	**Odds ratio (95% confidence interval)**	***P***	**Odds ratio (95% confidence interval)**	***P***
Age (per year)	1.01 (1.00–1.03)	0.147	–	–
Sex (male versus female)	1.14 (0.65–2.01)	0.643	–	–
Smoking	0.88 (0.56–1.39)	0.589	–	–
Hypertension	0.80 (0.51–1.25)	0.319	–	–
Type 2 diabetes	1.22 (0.78–1.93)	0.381	–	–
Hyperlipidemia	0.76 (0.48–1.20)	0.231	–	–
Hemoglobin (per g/dL)	0.87 (0.79–0.97)	0.010	–	–
Proteinuria				
Nil	1.00			
Trace	0.92 (0.48–1.78)	0.816	–	–
Positive	1.70 (0.87–3.31)	0.119		
Body weight (per kg)	1.00 (0.98–1.01)	0.659	–	–
Systolic blood pressure at admission (per 10 mmHg)	0.92 (0.86–1.00)	0.045	0.92 (0.85–1.00)	0.041
Diastolic blood pressure at admission (per 10 mmHg)	0.93 (0.82–1.06)	0.264	–	–
Heart rate at admission (per 10 beats/minute)	1.11 (1.00–1.23)	0.044	1.13 (1.02–1.25)	0.022
Chronic kidney disease status				
Stage 1	1.00		1.00	
Stage 2	1.64 (0.68–3.98)	0.275	1.68 (0.69–4.10)	0.252
Stage 3	1.57 (0.63–3.92)	0.332	1.56 (0.62–3.90)	0.346
Stage 4	4.15 (1.44–11.94)	0.008	3.68 (1.27–10.70)	0.017
Stage 5	3.73 (1.19–11.65)	0.024	3.43 (1.08–10.82)	0.036

An estimated glomerular filtration rate (eGFR) ≥ 90 mL/min/1.73 m^2^ is defined as stage 1, eGFR 60–89 mL/min/1.73 m^2^ is defined as stage 2, eGFR 30–59 mL/min/1.73 m^2^ is defined as stage 3, eGFR 15–29 mL/min/1.73 m^2^ is defined as stage 4, and eGFR < 15 mL/min/1.73 m^2^ is defined as stage 5.

## DISCUSSION

In this hospital-based retrospective observational study, we found that the prevalence of CKD stages 3 or above was 42.9% in patients with acute STEMI. The Global Registry of Acute Coronary Events revealed that about one third of patients presenting with STEMI or non-STEMI had CKD [9]. The high prevalence of CKD in Taiwanese STEMI patients might be explained by the higher prevalence of CKD in the Taiwanese population, which was reported to be 11.9% (6.9% at stages 3 to 5) [10].

A major finding of this study was that STEMI patients with CKD stage 4 and stage 5 were more likely to have multivessel significant stenosis. This finding is compatible with previous studies on the severity of coronary artery atherosclerosis in patients with CKD and acute coronary syndrome [8, 11]. Our study also found that older age, male sex, type 2 diabetes mellitus, and hyperlipidemia were independently associated with an increased risk of multivessel significant stenosis in patients with acute STEMI. These associations are consistent with the existing literature on traditional cardiovascular risk factors. In addition, systolic blood pressure at admission was found to be inversely associated with the risk of multivessel significant stenosis in patients with acute STEMI. A possible reason is that multivessel disease in AMI could induce large area of myocardial ischemia and left ventricular dysfunction, which might lead to a lower systolic blood pressure.

The association between renal function impairment and the number of coronary vessels with total occlusion in acute STEMI patients has rarely been reported. In this study, we found multivessel total occlusion was significantly associated with stage 4 and stage 5 CKD, after adjusting for systolic blood pressure at admission and heart rate at admission. This is a novel finding of our study, and is compatible with the finding that a higher proportion of CABG recipients in advanced CKD patients were noted in this study and in a previous report [12].

Patients with CKD may have a higher prevalence and longer exposure of traditional risk factors of coronary artery disease, such as older age, hypertension, type 2 diabetes mellitus, hypercholesterolemia, and obesity, than patients with normal renal function. In our study, An estimated glomerular filtration rate (eGFR) ≥ 90 mL/min/1.73 m^2^ is defined as stage 1, eGFR 60–89 mL/min/1.73 m^2^ is defined as stage 2, eGFR 30–59 mL/min/1.73 m^2^ is defined as stage 3, eGFR 15–29 mL/min/1.73 m^2^ is defined as stage 4, and eGFR < 15 mL/min/1.73 m^2^ is defined as stage 5.

CKD stages remained to be significantly associated with multivessel total occlusion even after adjusting for these risk factors. It has been documented that CKD patients are at higher risks of developing chronic inflammation, oxidative stress, and mineral metabolism disturbance [13]. Pro-inflammatory cytokines (tumor necrosis factor-α, interleukin-6, monocyte chemotactic protein-1, etc.) and inflammatory markers (e.g. C-reactive protein) can be elevated as the renal function deteriorates [14]. A cohort study on the progress of inflammation and oxidative stress in patients with CKD revealed that eGFR was inversely associated with malondialdehyde and associated with superoxide dismutase and glutathione peroxidase at baseline. After 12 months of follow-up, a higher level of serum creatinine was associated with increases in high sensitivity C-reactive protein and malondialdehyde [15]. These factors could be associated with accelerated atherosclerosis and might lead to involvement of multiple coronary arteries in patients with CKD.

Disturbances in mineral metabolism, such as hyperphosphatemia, hypocalcemia, hyperparathyroidism, and vitamin D deficiency, often develop in the early stage of CKD and manifest as the disease progresses to severe CKD. The management of mineral metabolism disturbance in CKD has been difficult. As a result, the development of hyperparathyroidism, hypercalcemia, hyperphosphatemia, and high calcium-phosphate production might cause calcification of heart valves and coronary vessels, which could increase mortality risk in CAD patients receiving hemodialysis [16].

In addition, chronic inflammation is associated with coronary artery calcification, which is evidenced by the association between C-reactive protein level and coronary artery calcification in patients with end-stage renal disease (ESRD) on peritoneal dialysis [17]. In ESRD, dialysis-related infection and other factors might lead to chronic inflammation. The characteristic of coronary artery calcification in severe CKD was the deposition of calcium over media layer of the coronary artery. As the severity of CKD increases, atherosclerosis of the intima layer and calcification of media layer would progress, which could increase the risk of cardiovascular mortality in patients with ESRD [18]. The Dallas Heart Study also reported that coronary artery calcification was more prevalent in patients with CKD at stages 3 to 5 compared with patients without CKD [19]. In addition, plaque components of coronary culprit lesions had been found to change from necrotic core-rich to calcium-rich plaques when renal function declined [20]. The increasing plaque burden of coronary artery in later stage CKD might explain the increasing number of coronary vessels with total occlusion observed in patients with early to advanced CKD stages in our study.

The intervention of acute STEMI in CKD can be challenging in cases with multivessel coronary artery disease, especially when increased risks of cerebrovascular events and bleeding complications need to be taken into consideration [21]. In AMI patients undergoing primary PCI, the 30-day and 1-year death rates, infarct artery reocclusion rate, and bleeding risk were higher in CKD than in non-CKD patients [22]. A study in patients undergoing primary PCI for acute STEMI showed that the prevalence of chronic total occlusion in a non-infarct-related artery was 13% in patients with CKD (defined as an eGFR <60 mL/min/1.73 m^2^), compared to 7% in those without [23]. Our study further found an increasing proportion of multivessel total occlusion (two or more vessels) from 3.9% in stage 1, 6.2% in stage 2, 6.1% in stage 3, 14.4% in stage 4, to 13.2% in stage 5 CKD among patients with acute STEMI. When patients with CKD progress to stages 4 and 5, more aggressive screening and treatment for coronary artery disease might be necessary.

In this study, patients who fit the inclusion criteria were recruited consecutively, which could minimize selection bias. All the patients included in the study had received coronary angiography, and therefore misdiagnosis of STEMI was unlikely. The large number of patients allowed us to obtain stable risk estimates even after adjusting for multiple risk factors. Nonetheless, this study has some limitations. We did not evaluate the effects of mineral metabolism disturbance. They are not routinely monitored in patients with acute STEMI, and therefore further studies investigating these factors are warranted. In addition, we did not study the effects of history of angina pectoris, myocardial infarction, or coronary intervention. They may be associated with multivessel involvement in STEMI patients, but the information was not available in all acute STEMI patients, especially those who were unconscious on arrival and never regained consciousness before death. Long-term follow-up studies are needed to address these issues.

In conclusion, in patients with acute STEMI, severe renal function impairment (CKD stage 4 and stage 5) was associated with a significant higher risk of having multivessel significant stenosis after adjusting for age, sex, type 2 diabetes mellitus, hyperlipidemia, and systolic blood pressure at admission. In addition, severe CKD (stages 4 and 5) was associated with a significant higher risk of having multivessel total occlusion after adjusting for systolic blood pressure and heart rate at admission. Clinicians should be vigilant to this phenomenon in the management of patients with CKD.

## MATERIALS AND METHODS

We conducted a study using the electronic patient record system of a regional teaching hospital in southern Taiwan. Records of consecutive patients with acute STEMI admitted to the hospital between March 1999 and October 2013 were reviewed. Patients who did not receive coronary angiography or had missing data in their records were excluded.

The study protocol was approved by the Institutional Review Board of the Ditmanson Medical Foundation Chia-Yi Christian Hospital, Taiwan (No. 100061). The Institutional Review Board waived the requirement for obtaining informed consent from the patients. All patient records were de-identified prior to analysis.

The diagnosis of STEMI was based on (1) concurrence of chest pain, symptoms compatible with acute heart failure, or unexplained syncope, (2) ST-segment elevation ≥ 1 mm in two inferior or lateral leads, or ≥ 2 mm in ≥ 2 precordial leads, and (3) elevation of creatine kinase-MB or troponin-I.

Serum creatinine was measured by the enzymatic method using an Accuras Auto Cre diagnostic kit (Shino-Test Corporation, Tokyo, Japan) with a Labospect 008 clinical analyzer (Hitachi High-Technologies Corporation, Tokyo, Japan) [24]. We calculated the estimated glomerular filtration rate (eGFR) using the Chronic Kidney Disease Epidemiology Collaboration (CKD-EPI) [25] as follows:

GFR = 141 &times; min(Scr/κ,1)^α^ &times; max(Scr/κ, 1)^−1.209^ &times; 0.993^age^ &times; 1.018 [if female] &times; 1.159 [if black]

Scr is serum creatinine (mg/dL), κ is 0.7 for females and 0.9 for males, α is −0.329 for females and −0.411 for males, min indicates the minimum of Scr/κ or 1, and max indicates the maximum of Scr/κ or 1.

The stage of CKD was determined according to the following definition:

Stage 1: eGFR ≥ 90 mL/min/1.73 m^2^ and clinical proteinuria, hematuria, or pathological evidence of renal parenchymal injury for 3 months.

Stage 2: eGFR between 60 and 89 mL/min/1.73 m^2^ and clinical proteinuria, hematuria or pathological evidence of renal parenchymal injury for 3 months.

Stage 3: eGFR between 30 and 59 mL/min/1.73 m^2^.

Stage 4: eGFR between 15 and 29 mL/min/1.73 m^2^.

Stage 5: eGFR < 15 mL/min/1.73 m^2^.

We evaluated coronary lesions using coronary angiography evaluation criteria [26]. The arteries of the coronary circulation system studied included the left main coronary artery, left anterior descending artery, left circumflex artery, and right coronary artery. We defined significant coronary stenosis as luminal stenosis of 50% or more, and multivessel involvement as the involvement of at least two of the above mentioned coronary arteries.

### Statistical analysis

We presented continuous variables as mean and standard deviation, and categorical variables as frequency and percentage. Analysis of variance and Chi-square test were used to compare the demographic and clinical characteristics of study patients with different CKD stages for continuous and categorical variables, respectively. Univariate and multiple logistic regression analyses with a backward elimination procedure based on the likelihood ratio test were conducted to assess the factors associated with multivessel significant stenosis and multivessel total occlusion. The possible two-way age and sex interaction effects with CKD stage in the multiple logistic regression models were tested. A *P* < 0.05 was considered statistically significant. All statistical analyses were performed using IBM SPSS Statistics for Windows, Version 23.0 (IBM Corp., Armonk, NY, USA).
